# Video-assisted anal fistula treatment (VAAFT) combined with ozonide oil dressing: standardization of technique in pediatric patients

**DOI:** 10.1007/s00464-024-10759-1

**Published:** 2024-03-05

**Authors:** Ciro Esposito, Giuseppe Autorino, Mariapina Cerulo, Fulvia Del Conte, Vincenzo Coppola, Giovanni Esposito, Annalisa Chiodi, Claudia Di Mento, Vincenzo Bagnara, Maria Escolino

**Affiliations:** 1 Division of Pediatric Surgery, Federico II University Hospital, Via Pansini 5, 80131 Naples, Italy; 2 CEINGE Advanced Biotechnologies, Naples, Italy; 3 Department of Pediatric Surgery, Policlinico G.B. Morgagni, Catania, Italy

**Keywords:** Anal fistula, Perianal abscess, VAAFT, Children, Ozonide, Dressing

## Abstract

**Background:**

Anal fistula and perianal abscess are commonly acquired anorectal pathologies in children. Surgical treatment options commonly adopted are fistulotomy, fistulectomy, cutting seton placement, and more recently video-assisted anal fistula treatment (VAAFT). Optimal postoperative wound dressing remains debated. This study aimed to report our series of pediatric patients, who received VAAFT and postoperative wound dressing using ozonide oil.

**Methods:**

All patients who underwent VAAFT between August 2018 and May 2023 were included in the study. Demographics, clinical features, pre-operative imaging, surgical details, outcome, and mid-term outcome data were retrospectively reviewed for each patient. All VAAFT procedures were performed under general anesthesia and using a 10-Ch fistuloscope.

**Results:**

Thirty-three VAAFT procedures were performed in 30 patients over the study period. The median patient age was 5.7 years (range 1.75–14). Anal fistula was idiopathic in 26/30 (86.6%), iatrogenic in 2/30 (6.7%), and secondary to Crohn’s disease in 2/30 (6.7%). The median duration of surgery was 23 min (range 18–40). All patients received ozonide oil dressing twice a day for 5 weeks postoperatively. The median hospital stay was 24 h (range 9–36). The median healing time was 28 days (range 17–39). With a median follow-up of 2 years (range 0.5–5), disease recurrence occurred in 3/30 (10%) patients with idiopathic fistula, who were re-operated using the same technique, with no further recurrence. No fecal incontinence or soiling was observed.

**Conclusion:**

Our series confirmed that VAAFT is a safe and effective technique to treat children with perianal fistula. The technique is versatile, allowing to treat fistulae of different etiologies. Postoperative course was painless and fast. Future comparative prospective studies are needed to better establish these conclusions.

**Supplementary Information:**

The online version contains supplementary material available at 10.1007/s00464-024-10759-1.

Anal fistula and perianal abscess are commonly acquired anorectal pathologies in infants and children [1]. Regarding the gender prevalence, there is a male preponderance. The most prevalent type is represented by idiopathic cryptoglandular fistula, which develops in the glands of the anal crypts. This condition is identified by a primary internal opening in the anal canal, a fistulous tract, and a secondary perineal opening accompanied by a discharge of purulent material [2]. They can be spontaneous because of cryptitis or can develop as surgical complication or secondary to anal/perianal trauma [3]. Parks classified anal fistula in 4 types: inter-sphincteric, trans-sphincteric, supra-sphincteric, and extra-sphincteric [4]. Regarding the clinical onset, idiopathic cryptoglandular fistulae may develop since the first month after birth until 2 years of age. Conversely, fistulae secondary to Crohn’s disease usually appear later in the adolescence period [5, 6].

Perianal abscesses in children require prompt treatment by incision and drainage under local anesthesia with low recurrence of perianal sepsis [7]. The comprehensive treatment of the fistulous tract can be deferred to a subsequent stage. The main objective is to manage the infection without compromising anal continence [2]. Surgical treatment options available include fistulectomy, fistulotomy, cryptotomy, cutting seton, or draining seton placement [8, 9]. Primary fistula treatment demonstrated better clinical efficacy in promoting the healing rate and decreasing the formation of fistulae in perianal abscesses in children compared with drainage alone [10]. Nevertheless, these techniques have been associated with high postoperative morbidity and high risk of disease recurrence and fecal incontinence [11]. According to the literature reports, the rate of disease recurrence after standard surgical treatment was about 20% [12]. Furthermore, postoperative fecal incontinence was reported in up to 25% of patients treated with cutting seton placement [13, 14]. Similarly, placement of draining seton was associated with disease recurrence in 7% and need for additional procedures in 27% of treated patients [9].

Treatment alternatives have been described to fasten the healing of both internal opening and fistula track, such as injection of platelet-rich fibrin sealant or fibrin glue and use of fistula plugs or ligatures [15–18]. Despite extensive evaluation of the therapeutic modalities, no clear consensus exists as to what is the gold standard approach.

Minimally invasive treatment of anal fistula was first reported in adults by Meinero, who described the video-assisted anal fistula treatment, also known as VAAFT [19]. This technique involves the identification and safe closure of the internal opening and the cauterization of the fistula track using a specially designed fistuloscope. While the technique is well established and widely used in adults, its standardization in pediatric patients is not as well established.

Another debated point with unclear effects on postoperative outcomes remains the optimal postoperative wound care. Studies have shown that pain during dressing changes is the most painful part of the wound recovery process for many patients [20].

In an earlier study, our group introduced the application of ozonide oil as wound dressing for patients with pilonidal sinus disease undergoing pediatric endoscopic pilonidal sinus treatment (PEPSiT). The outcomes revealed significant benefits, including accelerated healing and reduced likelihood of disease recurrence [21]. Drawing from such positive outcomes, we opted to apply the same dressing protocol for children with anal fistula undergoing VAAFT.

The aim of this study was to report a pediatric case series treated with VAAFT and postoperative wound dressing using ozonide oil.

## Materials and methods

All patients who underwent VAAFT between August 2018 and May 2023 in two divisions of Pediatric Surgery were enrolled in the study. Exclusion criteria were patients who had already received primary surgery for anal fistula, such as fistulotomy, fistulectomy, and cutting seton placement. Inclusion criteria encompassed all patients who underwent VAAFT, whether it was the primary procedure or a secondary intervention after prior incision and drainage of perianal abscess.

Surgical contraindications were fistula with recent inflammation (immature track), pelvic fistula (diagnosed by magnetic resonance), and no active discharge for at least 2 months.

Demographics, clinical features, pre-operative imaging, surgical details, postoperative outcome, and medium-term follow-up data were retrospectively reviewed for each patient. Postoperative pain was assessed using the Visual Analogue Scale (VAS). To enhance the reliability of pain assessment in infants and young children, an independent observer applied the Visual Analogue Scale (VAS). Conversely, older children, who were able to collaborate, actively participated in evaluating their postoperative pain using the VAS.

The study received appropriate Institute Review Board (IRB) approval.

### Operative technique

**Fig. 1:**
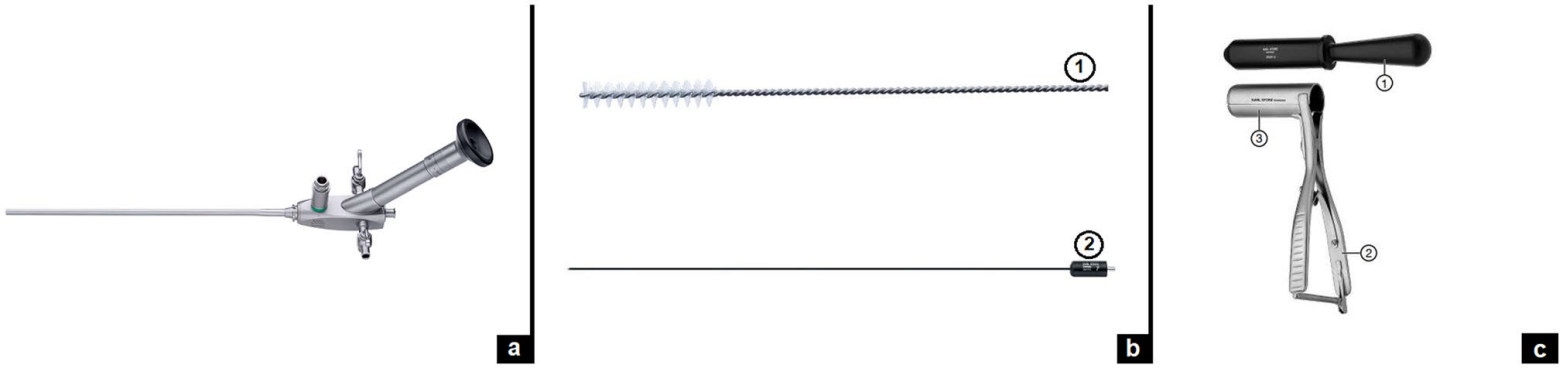
VAAFT equipment: fistuloscope (**a**), instruments (**b**) including brush (1) and monopolar electrode (2), and anal distending speculum (**c**) with obturator (1), handle with ratchet (2), and distending blades (3)

All procedures were performed under general anesthesia. The patient was placed in lithotomy position with 15°–20° Trendelenburg tilt. Infants and small babies were preferentially placed in a frog legs position. The equipment necessary for performing VAAFT included a 10-Ch fistuloscope along with a set of instruments comprising fistula brush, monopolar coagulation electrode, and an anal distending speculum for anal examination (Fig. 1). The anal distending speculum is adopted in conjunction with an obturator, a handle with a ratchet and distending blades. It allows for atraumatic insertion into the rectum, providing access to the area under examination or the operating field during the procedure. With a diameter of 3 cm, it is suitable for use in older children. The fistuloscope features an 8° direction of view and includes a straight working channel that also serves as irrigation channel. It has an operative length of 18 cm and an outer diameter of 3.3 × 4.7 mm. Glycine-mannitol 1% solution was adopted as irrigation solution for fistuloscopy. The VAAFT technique aims to destroy the fistula track while preserving the external anal sphincter.

We meticulously examined the video recordings of all VAAFT procedures and established a standardized technique comprising 5 separate steps:

1. *Initial diagnostic assessment*. This involves careful examination with a metallic probe of perianal area and perineum for external fistula opening(s). Using the anal distending speculum, careful assessment of the distal rectum is performed to localize the site of the internal opening of the fistula. When faced with challenges in identifying the fistula internal opening by probing, injecting hydrogen peroxide, methylene blue, or more recently, indocyanine green (ICG) through the external orifice may assist in visualizing the internal opening in most cases.
2. *Fistuloscopic exploration of the fistula track*. To facilitate the smooth introduction of the scope, which has an outer diameter of 3.3 × 4.7 mm, it is imperative that the external opening of the fistula is at least 5 mm in size. In cases where the external fistula opening is narrow, a gradual dilation using progressively larger probes is performed until reaching the required diameter. If needed, fibrous scar tissue is excised to enlarge the opening and allow inserting the fistuloscope. Prior to insertion, the tip of the fistuloscope is placed just within the external opening to allow hydrodistention and delineation of the fistula track.
3. *Identification of the internal fistula opening and/or secondary tracks*. Fistuloscopy is performed by advancing slowly the fistuloscope through the fistula track. This allows to visualize the main course of the fistula and identify all cavitations or secondary side tracts. At this stage, an attempt is made to identify the position of the internal fistula opening. After insertion of anal retractor, a jet of irrigation solution is seen exiting from the internal opening within the anal canal in some cases. In other patients, the internal opening may be partially obliterated. In such patients, the trans-illumination effect of the light of the fistuloscope through the bowel wall may be helpful to locate the internal opening. If the internal opening cannot be identified, no attempt should be made to create an artificial internal opening. Once the internal fistula opening has been identified, it is marked by placing 1 to 3 4/0 polyglactin stay sutures, which are placed through the full thickness of the rectal mucosa. The tails of the sutures are kept long and held outside the anal canal.
4. *Fulguration of the fistula track under direct vision*. Granulation tissue within the fistula track is initially removed using the fistula brush, which is inserted through the working channel of the fistuloscope. Fulguration of the entire lining of the fistula tract is carried out under direct fistuloscopic view by means of either flexible monopolar electrode or diode laser fiber, according to the equipment’s availability. Secondary tracts, if present, are entered with the fistuloscope and their walls are fulgurated.
5. *Closure of the internal fistula opening.* The anal retractor is re-inserted, thus providing a good view of the internal fistula opening with stay sutures. Mucosal closure of the internal opening is performed using either 4/0 polyglactin interrupted stitches or purse-string suture or a mucosa flap. A temporary dressing is applied to the external opening, after coagulating it with monopolar diathermy.

Video 1 reproduces VAAFT operative technique.

### Postoperative management

**Fig. 2:**
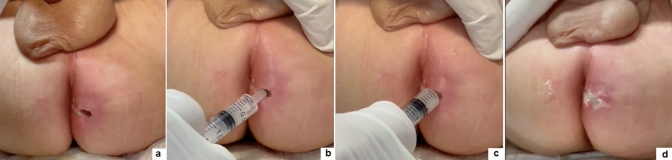
Wound dressing after VAAFT—The wound is washed with saline (**a**), then a syringe is inserted through the external opening (**b**), and 0.3–0.5 mL of ozonide oil is directly injected into the fistula track (**c**, **d**)

Antibiotic therapy (ampicillin 50 mg/kg) is given for 5 days postoperatively. Patients are discharged after overnight hospitalization to instruct parents about the wound care needed at home. The wound is washed with saline and then 0.3–0.5 mL of ozonide oil is directly injected into the fistula track by means of a syringe inserted through the external opening (Fig. 2). Finally, the wound is covered with wet gauze, avoiding use of adhesive tape on the perianal skin. We advise parents/caregivers to change the ozonide dressing 2 times per day and after any evacuation until complete healing is achieved. Patients are followed up at 1 week, 1 month, 3 months, 6 moths, 1 year postoperatively, and whenever recurrence of symptoms needs care.

### Statistical analysis

Descriptive data were reported as absolute rates and percentages for qualitative data. Median and range were used to describe quantitative variables.

## Results

Thirty-three procedures were performed in 30 patients over the study period. The male/female ratio was 23/7 (3.3). The median patient age was 5.7 years (range 1.75–14). Anal fistula was idiopathic in 26/30 (86.6%), iatrogenic in 2/30 (6.7%), who had previously received anorectal surgery for Hirschsprung’s disease, and secondary to Crohn’s disease in 2/30 (6.7%).

Complex fistulae, defined as those with more than a single tract connected to the internal opening, were identified in 3/30 (10%) patients.

VAAFT served as the initial surgical procedure in 21/30 (70%), while it was employed as secondary surgery following prior incision and drainage of perianal abscess in the remaining 9/30 (30%).

**Fig. 3:**
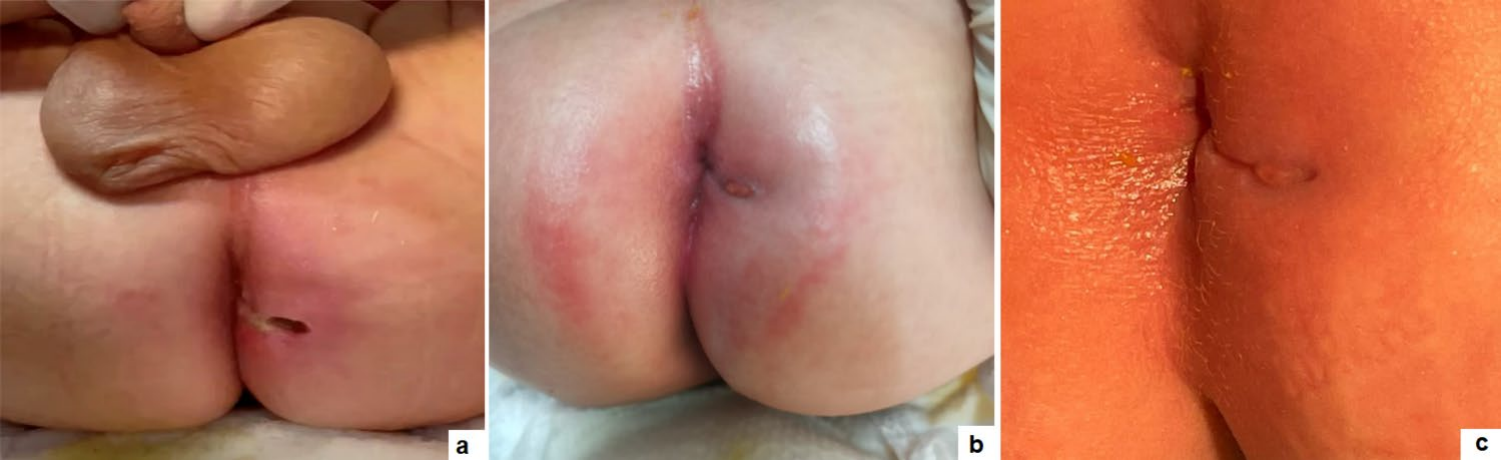
Wound healing at 1 day (**a**), 1 week (**b**), and 2 weeks (**c**) postoperatively

The median duration of surgery was 23 min (range 18–40). No intraoperative complications occurred. All patients received ozonide oil dressing twice a day for median 4 weeks postoperatively. The median postoperative pain score, assessed using VAS [score range 1–10], was 3.2 (range 1–5). Pain therapy was limited to the first postoperative day with intravenous paracetamol 15 mg/kg 8 hourly for all inpatients. None of the patients required supplementary pain therapy. No postoperative complications were observed. The median hospital stay was 24 h (range 9–36). The median healing time was 28 days (range 17–39) (Fig. 3).

At median follow-up of 2 years (range 0.5–5), disease recurrence occurred in 3/30 (10%) patients with idiopathic fistula, who were re-operated using the same VAAFT technique, with no further recurrence. No fecal incontinence or soiling was observed.

Patient characteristics and outcomes of VAAFT are summarized in Table 1.

**Table 1:** Patient characteristics and outcomes of VAAFT

Patients, *n*	30
M/F, *n/n*	23/7 (3.3)
Median patient age, years (range)	5.7 (1.75–14)
Type of fistula:	2 (6.7%)
Idiopathic, *n* (%)	
Iatrogenic, *n* (%)	2 (6.7%) surgery for Hirschsprung
Secondary to Crohn’s disease, *n (%)*	26 (86.6%)
Total VAAFT procedures, *n*	33
Median operative time*, min (range)*	23 (18–40)
Intraoperative complications, *n* (%)	0
Median postoperative VAS pain score, *n* (range)	3.2 (1–5)
Postoperative complications, *n* (%)	0
Median hospital stay, hours (range)	24 (9–36)
Median follow-up, years (range)	2 (0.5–5)
Disease recurrence, *n* (%)	3 (10%)
Re-operation, *n* (%)	3 (10%)

## Discussion

Surgical procedures for anal fistula have 3 main objectives: identification of fistula’s track and its internal opening, excision of fistula’s track, and preservation of anal sphincter function. Traditional surgical procedures for anal fistula included fistulotomy for superficial fistulae, fistulectomy for complex and deep fistulae, and staged fistulotomy with cutting seton placement for high fistulae [5, 7]. These procedures were associated with high postoperative morbidity, long time of wound healing, and need for regular painful dressings change [11]. Fecal incontinence was a significant complication in these patients, especially in complex and recurrent fistulae [13, 14]. Placement of draining seton alone was described as a viable treatment option for definitive symptomatic management of anal fistulae [9]. However, this treatment option was associated with disease recurrence in 7% and need for additional procedures in 27% of treated patients [9].

The video-assisted anal fistula treatment (VAAFT), first described in adults by Meinero and Mori in 2011 [19] and in pediatrics by Pini Prato in 2016 [22], relies on the principles of excision, cleaning, and fulguration of the fistula’s main and secondary tract(s) and identification and safe closure of the internal fistula opening to achieve the complete healing of the fistula. The innovation of the VAAFT is the precise identification of the fistula anatomy and the internal fistula opening using the fistuloscope and the fulguration of the fistula’s tract walls under direct vision using a monopolar electrode. There are no surgical incisions since surgical access is obtained via the pre-existing external opening of the fistula. The 10-Ch fistuloscope with 8° viewing angle allows to identify all side tracts of the fistula and any endoanal or endorectal orifices. Closure of the internal fistula opening is an essential step of the procedure to dramatically reduce the risk of disease recurrence. Pini Prato et al. [22] reported 100% success rate for those patients who underwent a complete VAAFT. Conversely, persistent anal fistula was observed in two patients who underwent diagnostic fistuloscopy without complete VAAFT and recurrence in one of the patients who underwent fistuloscopy and electrocoagulation without mucosal sleeve closure of the endorectal opening. Visualization of the fistula tract and its branches facilitates the precise identification of the course of the fistula and its internal opening. This capability is crucial in preventing the inadvertent creation of a false tract or false internal opening that may occur when blindly and forcefully exploring the tract with a fistula probe.

Closure of the internal opening in VAAFT can be accomplished using various methods, such as a mucosal sleeve, interrupted stitches, or a purse-string suture. The selection of the method is primarily depending on the surgeon’s preference and anatomic factors. If the orifice is narrow or partially concealed, interrupted stitches, or a purse-string suture might be adequate for closure. In cases with large or multiple internal openings, a mucosal sleeve may be necessary. We did not observe any difference between the two methods in the outcomes.

When faced with challenges in identifying the fistula internal opening by probing, employing the hydrodistension effect of the fistula tract and using trans-illumination with the light from the fistuloscope passing through the bowel wall can be beneficial. Alternatively, injecting hydrogen peroxide, methylene blue, or more recently, indocyanine green (ICG) through the external orifice may assist in visualizing the internal opening in most cases. If these methods, including trans-illumination, prove ineffective in localizing the internal fistula opening, the recommended course of action is limited to the fulguration and debridement of the fistula tract.

The results of our series demonstrated that this technique is feasible and safe in children. It just requires a short admission. Additionally, VAAFT is versatile in its application, suitable for addressing both simple and complex fistulae. Also, Crohn’s anal fistulae can be effectively treated using this technique. The procedure can be successfully repeated in case of recurrent fistulae after VAAFT. Moreover, it can be utilized across a wide age range, from infancy to adolescence.

In our series, with a median follow-up of 2 years, the results were very satisfactory, leading to 90% success rate and 10% recurrence rate. A crucial issue in the surgical treatment of complex anorectal fistulae is to provide high rates of definitive closure without significant impairment of fecal continence. VAAFT allowed to avoid this issue. None of our patients developed postoperative fecal incontinence or soiling.

We believe that the standardization of the surgical technique is an essential factor contributing to the success of the procedure. Following meticulous examination of video recording of all VAAFT procedures, we established standardization of VAAFT in 5 separate steps: (1) Initial diagnostic assessment; (2) Fistuloscopic exploration of the fistula track; (3) Identification of the internal fistula opening and/or secondary tracks; (4) Fulguration of the fistula track under direct vision; and (5) Closure of the internal fistula opening. Regarding the learning curve, it can be shortened if the operating surgeon has previous experience with endoscopic surgery. In any case, we believe that at least 10–15 procedures are necessary to master the technique.

Another key point of the procedure is to use the specific technical equipment needed for VAAFT. It requires initial costs to purchase the VAAFT instrumentation. However, the fistuloscope and accessory instruments are reusable, thus the initial costs are likely to be recovered soon. Additionally, there are significant advantages to patients such as reduced pain, absence of perianal wounds, no needing for painful dressings, fast recovery, and early return to daily activities. Balaz et al. [11] reported a duration of hospitalization ranging from 2 to 30 days in different patient groups undergoing fistulotomy. Conversely, in our VAAFT series the median hospital stay was 24 h.

A crucial point for successful outcome of patients operated for anal fistula is the postoperative wound care. The key clinical priorities for postoperative care of high anal fistulae are to keep the perianal area clean, to ensure unobstructed drainage of the incision and to avoid bridge-type healing. Studies have shown that dressing changes was the most painful part of the wound recovery process in many patients [20, 23].

We proposed a simple method of dressing, which significantly reduced the pain of patients and was learned quickly by parents, reducing the time of hospitalization and the number of patients coming to the clinic for dressing change. The most important part of this dressing is the use of ozonide oil. The oil formulation allows the ozone to be stabilized in an olive oil matrix. Stable ozonides act on the wound healing through different pathways: antibacterial, antiviral, and antifungal action; carrier action of native O_2_; antioxidant action; modulation of the inflammatory phase; and promotion of angiogenesis [24, 25]. To increase the therapeutic effect, it is worth to inject the product directly within the fistula cavity. The rationale of intralesional application of the ozonide oil was based upon the observation that the ozonated composition exhibited the capability to deliver nascent oxygen deep within the lesion, promoting accelerated healing, while avoiding irritation to the skin and mucosa [26]. Our dressing method was painless as it avoided using adhesive tape or dry gauze which could stitch to the wound. The oil formulation was easier to manage and apply directly into the fistula track by inserting the tip of a syringe through the external orifice. This maneuver was painless, as observed during dressing change by either nurses or parents/caregivers. It is crucial that parents strictly adhere to the wound protocol. They must keep clean the perianal area and change the dressing after each evacuation until the completion of wound healing.

The main limitations of the present study are the relatively small series, the retrospective nature, and the lack of a control arm. Future prospective follow-up studies are necessary to better address the advantages and shortcomings and long-term efficacy of the technique.

Our series confirmed that VAAFT is a safe and effective technique to treat children with perianal fistula. The technique is versatile, allowing to treat fistulae of different etiologies. Postoperative course was painless and fast. Future comparative prospective studies are needed to better establish these conclusions.

### Supplementary Information

Below is the link to the electronic supplementary material.

Supplementary file1 (MP4 270498 KB)
